# Morphological and Functional Differences between Athletes and Novices in Cortical Neuronal Networks

**DOI:** 10.3389/fnhum.2016.00660

**Published:** 2017-01-04

**Authors:** Xiao-Ying Tan, Yan-Ling Pi, Jue Wang, Xue-Pei Li, Lan-Lan Zhang, Wen Dai, Hua Zhu, Zhen Ni, Jian Zhang, Yin Wu

**Affiliations:** ^1^School of Physical Education and Coaching, Shanghai University of SportShanghai, China; ^2^Shanghai Punan Hospital of Pudong New DistrictShanghai, China; ^3^Institutes of Psychological Sciences, HangZhou Normal UniversityHangzhou, China; ^4^School of Kinesiology, Shanghai University of SportShanghai, China; ^5^Division of Neurology, Krembil Neuroscience Centre and Toronto Western Research Institute, University Health Network, University of TorontoToronto, ON, Canada; ^6^School of Economics and Management, Shanghai University of SportShanghai, China

**Keywords:** basketball player, motor expertise, magnetic resonance imaging, plasticity, resting state functional connectivity

## Abstract

The cortical structural and functional differences in athletes and novices were investigated with a cross-sectional paradigm. We measured the gray matter volumes and resting-state functional connectivity in 21 basketball players and 21 novices with magnetic resonance imaging (MRI) techniques. It was found that gray matter volume in the left anterior insula (AI), inferior frontal gyrus (IFG), inferior parietal lobule (IPL) and right anterior cingulate cortex (ACC), precuneus is greater in basketball players than that in novices. These five brain regions were selected as the seed regions for testing the resting-state functional connectivity in the second experiment. We found higher functional connectivity in default mode network, salience network and executive control network in basketball players compared to novices. We conclude that the morphology and functional connectivity in cortical neuronal networks in athletes and novices are different.

## Introduction

Cortical plasticity is an intrinsic property of the human brain and occurs after long-term training under various conditions (Blakemore and Frith, 2005; Pascual-Leone et al., 2005). Structural differences were found in regional cortical morphology between musicians and non-musicians (Gaser and Schlaug, 2003). Interestingly, London taxi drivers have larger gray matter volume than that in healthy controls or even non-taxi drivers in posterior hippocampi where information of spatial representation is stored (Maguire et al., 2000). However, it is not clear whether long-term training may have effects on cortical morphology with plasticity in motor related cortical areas and whether these effects may contribute to the improvement in motor functions. Elite athletes in the confrontational sports (e.g., basketball etc.) start training since childhood. The sophisticated skills in confrontational sports are likely due to the involvement of different brain areas related to various cortical networks (di Pellegrino et al., 1992; Gallese et al., 1996). These elite athletes offer a special model for studying the long-term training related cortical plasticity with changes in multiple brain areas. In contrast to the long time period required for the longitudinal studies (or often nearly impossible due to extremely long time consumption), a cross-sectional paradigm (cohort paradigm) with a comparison between highly skilled elite athletes and novices was widely used recently (Imfeld et al., 2009; Jäncke et al., 2009; Wei et al., 2011; Fauvel et al., 2014). Although the cross-sectional paradigm may not be more sufficient than the longitudinal paradigm, the study using cross-sectional paradigm is more practicable and the results obtained from a cross-sectional designed study also provide an important indication of the presence of plasticity effects with long-term training. Voxel-based morphometry (VBM) is a neuroimaging analysis technique that allows investigation of focal differences in brain anatomy (Ashburner and Friston, 2000). In the present study, we compared the gray matter volumes in various brain areas in basketball players with those in novices in a cross-sectional paradigm by measuring the structural variation in cortical morphology with VBM. We hypothesize that the gray matter volumes in motor related cortical areas are different between basketball players and novices. In addition, since long-term training process leads to refined cognitive functions such as visual search (Williams et al., 1999; McRobert et al., 2007) and sensory perceptions (Aglioti et al., 2008; Wu et al., 2013) in elite athletes, we also expect that the morphological difference between basketball player and novices may be present in cortical areas responsible for cognitive functions.

Motor expertise involves several internal processes requiring organization and integration of sensory and motor information in different cortical areas (Lisberger, 1988). Neuroimaging studies have showed that the human brain is intrinsically organized into a set of spatially distributed, functionally specific networks (Damoiseaux et al., 2006; Bressler and Menon, 2010). Cortical plasticity with long-term training to gain motor expertise is complex. The interaction of cortical activation among different brain areas at the network level may be associated with multi-factorial process of cortical plasticity (Dosenbach et al., 2008). In particular, default mode network, salience network and executive control network are major functional networks relevant to motor and cognitive functions (Bressler and Menon, 2010; Cocchi et al., 2014). Default mode network is activated during motor related spontaneous cognition (Buckner et al., 2008; Mantini and Vanduffel, 2013). The salience network plays an important role in guiding orientation of attention and monitoring of errors during events with internal and external activities (Eckert et al., 2009). The executive control network is responsible for high-level cognitive functions during motor behaviors (Alvarez and Emory, 2006; Fox et al., 2006). Our second hypothesis is that the resting-state functional connectivity in basketball players and novices are different, as relatively less evidence was found in functional brain network in top athletes. We selected the cortical areas with larger gray matter volumes in basketball players (compared to novices, defined in the VBM analysis) as the seed regions and used a seed-based approach to test the functional connectivity in two subject groups. We predicted that the different resting-state functional connectivity between basketball players and novices will be related to the cortical areas located in default mode network, salience network and executive control network which are relevant to the motor and cognitive functions in the basketball players.

## Materials and Methods

### Participants

Twenty-one basketball players (mean age 21.3 ± 1.3 years, age range 18–23 years) and 21 novices (mean age 21.9 ± 0.8 years, age range 19–24 years) were studied. All subjects were male (Shanghai University of Sport is one of the major training centers for men’s basketball in China). The basketball players were national first-class athletes and were trained five sessions per week (each daily session about 3 h) for 10–15 years (mean duration, 11.4 ± 2.3 years). The novices were university students without professional training in basketball or any other sports. Basketball players were taller (190.6 ± 3.4 cm) than healthy controls (176.8 ± 2.9 cm; *t* = 14.1, *p* < 0.001). The experimental protocol was approved by the regional ethics committee of the Shanghai University of Sport. All subjects gave written informed consent in accordance with the Declaration of Helsinki.

### Magnetic Resonance Imaging

Imaging scanning was performed using a 3T Siemens scanner in the functional magnetic resonance imaging (fMRI) center at the East China Normal University. The anatomical difference in gray matter volume between basketball players and novices was tested using the T1-weighted structural image scanning. A high-resolution image with 192 slices was acquired using a 3-dimension fast-field echo sequence (echo time (TE) = 2.34 ms, repetition time (TR) = 2530 ms, flip angle (FA) = 7°, field of view (FOV) = 256 mm^2^ × 256 mm^2^, slice thickness = 1 mm, inversion time (TI) = 1100 ms). The resting-state fMRI scanning was performed with a gradient echo planar imaging sequence (TE = 30 ms, TR = 2000 ms, FA = 90°, FOV = 240 mm^2^ × 240 mm^2^, slice thickness = 3 mm). A total of 210 scans were obtained from each subject. Subjects were instructed to keep themselves relaxed while lying still in the scanner with their eyes closed.

### Optimized Voxel-Based Morphometry Analysis

The imaging data analysis was performed using Statistical Parametric Mapping version 8 software^1^ implemented in MATLAB 7.4. As the basketball players were taller than novices and the brain volume varied with the height in individuals, we applied the optimized VBM approach (Good et al., 2001) to compare the gray matter volumes in two groups by creating a study-specific template. In the pre-processing step, each reoriented image was segmented (unified segmentation) into gray matter, white matter and cerebrospinal fluid (Ashburner and Friston, 2005). Segmented gray matter images of all subjects were rigidly transformed and averaged to create the study-specific template. The aligned gray matter images in each subject were normalized with the study-specific template. Modulation with Jacobian determinant was used to correct volume changes caused by spatial normalization. The modulated images were finally transformed into the Montreal Neurological Institute space and smoothed with a 6-mm full-width at half maximum Gaussian kernel.

**Figure 1:**
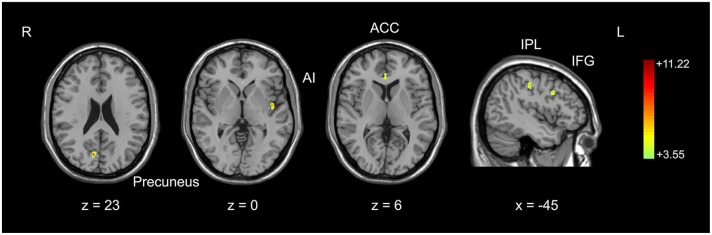
**Comparison of gray matter volume between basketball players and novices.** Cortical areas with more gray matter volume in basketball players compared to novices was shown. Color scale represents the significant *t*-values (corrected *p* < 0.05). L = left; R = right. Abbreviations for brain areas: ACC, anterior cingulate cortex; AI, anterior insula; IFG, inferior frontal gyrus; IPL, inferior parietal lobule.

Pre-processed gray matter images in basketball players and novices were compared with a two-sample *t*-test (second-level) run at the whole brain level. The height of subject was set as a covariate to exclude the potential contamination caused by different sizes of brain in two groups. The *t*-map was set at a corrected significance level of *p* < 0.05. AlphaSim correction (REST_V1.8) with Monte Carlo simulation was used to correct for multiple comparisons (Poline et al., 1997; Song et al., 2011) with consideration both for the individual voxel probability and cluster size threshold. Based on the results of optimized VBM analysis, brain areas with larger gray matter volume in basketball players were identified (Figure 1). We also tested the correlation between gray matter volumes in these areas and training time in basketball players with the Pearson correlation coefficient. These areas were further selected as the seeds for the subsequent functional connectivity analyses. The seed was defined as a 6 mm radius sphere.

### Functional Connectivity Analysis

**Figure 2:**
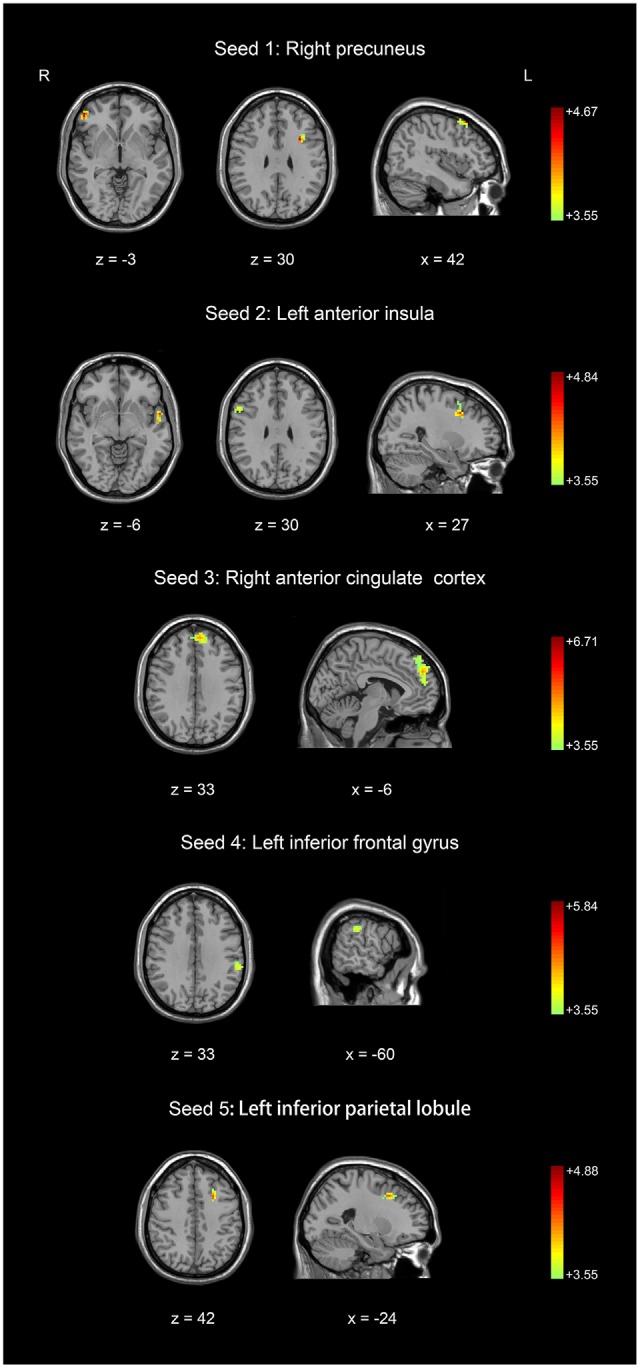
**Comparison of resting-state functional connectivity between basketball players and novices.** Higher resting-state functional connectivity in basketball players compared to novices was shown. Five seed regions (three in the left brain and two in the right brain) were extracted. Color scale represents the significant *t*-values (corrected *p* < 0.05).

We defined five seeds (Figure 1) for the resting-state functional imaging analysis. The pre-processing for the resting-state functional imaging analysis included slice time correction, rigid body movement correction, normalization of the functional images by directly registering onto the Montreal Neurological Institute echo planar imaging template (interpolated spatial resolution 3 mm^3^ × 3 mm^3^ × 3 mm^3^) and spatially smoothing (6 mm full-width at half maximum). The voxel-wise correlation analysis was conducted after the initial imaging data were temporally filtered (0.01–0.08 Hz). The resting-state time series of the five selected seed regions were extracted using MarsBaR toolbox (Brett et al., 2002). The correlation coefficient (*r*-value) between the seed region (6 mm radius sphere) and other voxels of the whole brain (excluding those in the seed region) was computed. The correlation coefficient was converted into a z score by Fisher’s *r*-to-*z* transformation to generate a contrast matrix for each seed region in each subject. For the group data analysis, we used a two-sample *t*-test to compare the difference between basketball players and novices (Figure 2). The *t*-test was repeated five times (one *t*-test for each seed region). AlphaSim correction with Monte Carlo simulation was used to correct for multiple comparisons. The *t*-map was set at a corrected significance level of *p* < 0.05.

**Figure 3:**
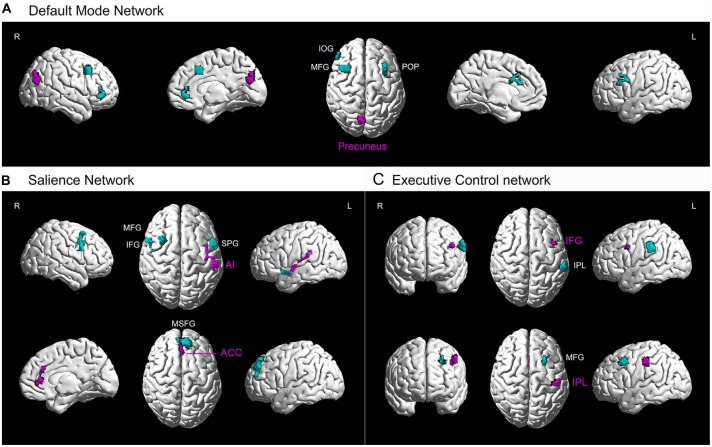
**Higher resting-state functional connectivity in basketball players related to three cortical networks.** Five seed regions (purple) with enhanced gray matter volume showed stronger functional connectivity with several brain areas (green) in the basketball players compared to novices. These functionally connected brain areas are parts of default mode network **(A)**, executive control network **(B)** and attention network **(C)**. L = left; R = right. Abbreviations for brain areas: ACC, anterior cingulate cortex; AI, anterior insula; IFG, inferior frontal gyrus; IOG, inferior orbitofrontal gyrus; IPL, inferior parietal lobule; MFG, middle frontal gyrus; MSFG, medial superior frontal gyrus; POP, pars opercularis (of the inferior frontal gyrus); STP, superior temporal pole.

One purpose of our study was to investigate the differences in functional connectivity between two groups as they were applied to the cortical networks with potential interests. We predicted the differences in functional connectivity between two subject groups would be due to different motor and cognitive functions in them which were likely related to different cortical networks. We specifically focused on three cortical networks, including the default mode network, salience network and executive control network. Therefore, we illustrated the data with functional connectivity from five seed regions (superimposed results of 5 *t*-tests) and projected the data onto a network brain template (Brain NetViewer^2^; Xia et al., 2013; Figure 3). The clusters of involved brain areas were extracted separately for each seed region and an ICBM152 brain template was used (REST_V1.8; Song et al., 2011). This three-dimensional volume-to-surface mapping provided more intuitive information about the seed regions and other functionally connected brain areas within three cortical networks and exhibited the spatial distribution of these cortical networks in the brain (Margulies et al., 2013).

## Results

### Gray Matter Volume

We used an optimized VBM technique to analyze T1-weighted anatomical scanning and set height as a covariate to correct for the potential effect of contamination caused by different brain sizes in two subject groups. It was found that basketball players had larger gray matter volumes than novices in multiple brain areas. These areas included right precuneus, left anterior insula (AI), right anterior cingulate cortex (ACC), left inferior frontal gyrus (IFG) and left inferior parietal lobule (IPL; Figure 1; Table 1). These areas were selected as the seeds for the functional connectivity analyses. The inverse contrast analysis did not show larger volumes in novices compared to players in any brain areas. No significant correlation was found between the gray matter volume and training time in five seed regions in basketball players.

**Table 1:** **Gray matter volumes in five seed areas in basketball players and novices (players > novices)**.

Brain region	Side	*x*	*y*	*z*	Voxels	*t*-value
Precuneus (BA 31)	R	11	−74	23	52	8.52
Anterior insula (BA 13)	L	−42	−6	−0	136	7.88
Anterior cingulate	R	2	32	6	35	6.32
cortex (BA 32)
Inferior frontal gyrus (BA 9)	L	−45	11	33	48	6.75
Inferior parietal lobule (BA 3)	L	−45	−24	44	75	7.50

*A, Brodmann’s area; L, left; R, right. Coordinates refer to Talairach space. Brain areas with corrected *p* < 0.05 were listed*.

### Resting-State Functional Connectivity

Five seed regions were connected with multiple brain areas at resting state both in basketball players and novices. Importantly, we found resting-state functional connectivity is different for basketball players and healthy controls in functional networks related to various brain areas (Figure 2; Table 2). Specifically, the right precuneus showed more resting connectivity with right inferior orbitofrontal gyrus (IOG), left pars opercularis (POP) of the IFG and right middle frontal gyrus (MFG) in the basketball players group. The connectivity between left AI and right MFG, right IFG, left superior temporal poles (STP) was stronger in the player group than that in the novice group. Right ACC was more functionally connected with left medial superior frontal gyrus (MSFG) in the basketball player group. Left IFG was more functionally connected with the left IPL while the left IPL was more functionally connected with the left MFG in the basketball player group compared to the novice group. On the other hand, the reversed comparison did not find stronger connectivity between seed region and any other cortical areas in novices compared to basketball players.

**Table 2:** **Resting-state functional connectivity between seed regions and other brain areas in basketball players and novices (players > novices)**.

Brain region	Side	*x*	*y*	*z*	Voxels	*t*-value
**Seed 1: Right precuneus**
Inferior orbitofrontal gyrus (BA 47)	R	51	45	−3	53	4.43
Inferior frontal gyrus (pars opercularis, BA 47)	L	−30	12	30	58	4.52
Middle frontal gyrus (BA 8)	R	42	18	57	49	3.89
**Seed 2: Left anterior insula**
Superior temporal pole (BA 38)	L	−57	3	−6	34	4.05
Middle frontal gyrus (BA 6/BA 8)	R	27	12	39	84	4.39
Inferior frontal gyrus (BA 9)	R	54	12	30	48	3.82
**Seed 3: Right anterior cingulate cortex**
Medial superior frontal gyrus (BA 9)	L	−6	51	33	167	4.96
**Seed 4: Left inferior frontal gyrus**
Inferior parietal lobule (BA 40)	L	−60	−33	33	31	4.15
**Seed 5: Left inferior parietal lobule**
Middle frontal gyrus (BA 8)	L	−24	15	42	25	3.78

*BA, Brodmann’s area; L, left; R, right. Coordinates refer to Talairach space. Brain areas with corrected p < 0.05 were listed*.

Furthermore, we projected the data onto a network brain template. It was confirmed that right precuneus had stronger connectivity with left POP of the IFG and right IOG, MFG in default mode network in basketball players compared to that in novices (Figure 3A). For the salience network (Figure 3B), left AI had stronger connectivity with left STP and right IFG while right ACC was more functionally connected with left MSFG in basketball players than novices. For the executive control network (Figure 3C), functional connectivity between left IFG and IPL and that between IPL and MFG was stronger in basketball players than that in novices.

## Discussion

We investigated the difference in brain structure and resting-state functional connectivity between basketball players and novices. The novel finding was that basketball players showed greater gray matter volume in five brain areas (Figure 1). Furthermore, basketball players displayed higher resting-state functional connectivity between these five seed regions and other cortical areas compared to novices. These cortical areas are located in default mode network, salience network and executive control network which are related to motor and cognitive functions in basketball players (Figures 2, 3).

### Gray Matter Volume

We found larger volume of gray matter in right precuneus, left AI, right ACC, left IFG and left IPL in basketball players compared to novices. Precuneus is associated with processing spatial information during motor execution and preparation (Kawashima et al., 1995; Cavanna and Trimble, 2006). In particular, precuneus was involved in target tracking tasks with special requirement for attention to spatial information (Wenderoth et al., 2005; Cavanna and Trimble, 2006). Regular practicing skill of tracking the frequently moving targets in basketball may explain the volume increase in precuneus in basketball players compared to novices. Insula shows high activation during complex behavioral tasks (Craig, 2009) and plays an important role in making a rapid decision in a risky situation (Craig, 2002; Singer et al., 2009). Such ability with superior motor related perceptual functions is required in basketball because the players often perceive their self-positioning in the court and make decision in offending/defending strategy (Bar-Eli and Tractinsky, 2000; Llorca-Miralles et al., 2013; Kinrade et al., 2015). Our results also confirmed that physical exercise induces volume increase in the ACC (Flöel et al., 2010; Prakash et al., 2010) and supported the opinion that ACC is the major neuronal substrate for attention (Osaka et al., 2007) and action selection (Rushworth, 2008). The IFG is related to the action observation and imitation (Buccino et al., 2004; Calvo-Merino et al., 2005, 2006; Iseki et al., 2008; Caspers et al., 2010), which may be essential in basketball because the programming and execution of motor plan in basketball highly relies on the action observation of the opponent players (Fujii et al., 2014a,b). The IPL is important for complex cognitive functions, including visual perception, spatial perception and visuomotor integration (Anderson, 2011). The present finding of more gray matter volume in IPL in basketball players was consistent with previous evidence from neuroimaging studies that IPL was activated during action observation (Grèzes and Decety, 2001; Buccino et al., 2004; Hamilton and Grafton, 2006; Chong et al., 2008) and anticipation with correct understanding of the movement (Rizzolatti et al., 2006).

The basketball players in the present study are top athletes in China. The expertise in basketball skill largely varies depending on the special role that the athlete plays on the court (e.g., point guard and center have completely different playing styles) although our subjects have a similar duration in basketball training. The diversity in playing style with similar training duration might explain why we did not find significant correlation between gray matter volume and training duration in basketball players. This is different from our recent transcranial magnetic stimulation study in badminton players whose motor cortical excitation and inhibition are correlated to training years (Dai et al., 2016).

### Functional Connectivity in Cortical Neuronal Networks

Independent component analysis and seed-based correlation analysis are two most common techniques used in functional connectivity data analysis (Biswal et al., 1995; Fox et al., 2005). Although network map obtained from the independent component analysis may be used as a reference to interpret the results from seed-based correlation analysis, two data analysis techniques are based on different mathematical models (Calhoun et al., 2001; van de Ven et al., 2004; Joel et al., 2011). Independent component analysis provides a means to test several spatially separated cortical networks at once. However, the value of a voxel being tested with the independent component analysis represents the correlation between the time series of this voxel and the mean time series of a particular cortical network. The interpretation for these data-driven networks largely depends on the predetermined number of components for production which changes the patterns of spatially separated cortical networks. The interpretation is further challenged by the complexity of noise identification process which is often determined with system selection by the user. On the other hand, seed-based correlation analysis requires the selection of the seed regions. Voxel value from seed-based correlation analysis reflects the degree to which the time series of a tested voxel is correlated with the time series of the seed region. Owing to inherent simplicity, high sensitivity and ease of interpretation, seed-based correlation analysis is widely used to test the functional connectivity between a given seed region and the other cortical areas. We defined five seed regions with larger gray matter volumes in basketball players through VBM analysis at the first step in our study. Therefore, we specifically tested whether the correlations of time series between the seed regions and other cortical areas were different in basketball players and novices. A seed-based correlation approach is likely better and more practicable to identify the difference between two groups in our study. In addition, it may be worth mentioning that previous studies reported similar results when same resting-state fMRI data set was processed by independent component analysis and seed-based correlation analysis techniques (Damoiseaux et al., 2006).

Default mode network includes precuneus, posterior cingulate, medial prefrontal cortex and inferior parietal cortex (Raichle et al., 2001; Fox et al., 2005). We found greater connectivity between precuneus and medial prefrontal cortex in basketball players, supporting the functions of precuneus and the medial prefrontal cortex as the core nodes in default mode network (Martinelli et al., 2013). Our results were consistent with previous study performed in musicians that long-term motor learning and expertise experience lead to resting-state functional connectivity changes in the default mode network (Fauvel et al., 2014). As precuneus and medial prefrontal cortex highly involve in self-related episodic memory (Dörfel et al., 2009), it may be explained that higher activity in the default mode network is caused by frequently processed self-related episodic memory in basketball playing.

Salience network is composed of AI, dorsal ACC (dACC) and ventrolateral prefrontal cortex (Seeley et al., 2007; Chan et al., 2008). We found high connectivity between AI, frontal cortex and STP in basketball players. The result may be consistent with the notion that AI is highly involved in extracting key salient stimuli from multiple inputs (Menon and Uddin, 2010). Our previous study also reported greater AI activity when basketball players noticed incorrect anticipation during observation of a basketball free throw (Wu et al., 2013). Frontal cortex is responsible for the episodic memory extraction (McDermott et al., 1999; Wagner, 1999; Lepage et al., 2000; Cabeza et al., 2002) while STP is related to the storage of semantic memory (Markowitsch, 1995; Simmons and Martin, 2009). Our results support the idea that memory extraction and storage are essential in basketball. We also found strong connectivity between ACC and MSFG (one part of frontal cortex) in basketball players. As ACC is related to the detection and processing of salient information and monitoring of errors (Kiehl et al., 2000; Hester et al., 2005; Etkin et al., 2011), our results may suggest that the process of semantic memory with interaction between ACC and frontal cortical area is important to maintain the high performance for the basketball players.

Executive control network is distributed in the frontoparietal system which comprises the dorsolateral prefrontal cortex and posterior parietal cortex. Particularly, frontal cortex is a primary region in modulating regular allocation of spatial attention (Schafer and Moore, 2011) and parietal cortex is heavily involved in spatial awareness (Behrmann et al., 2004). Our results that connectivity between the IFG, MFG and IPL is stronger in basketball players compared to novices may verify the idea that executive control network is the key structure for converting selective and spatial attention (Wu et al., 2007) in athletes with high motor expertise.

### Motor and Cognitive Functions in Basketball Players

Basketball is a confrontational sport with open motor skill in which movements and actions of the players largely depend on the understanding of the environment and actions of other players (both the team mates and the players on the opposite side; Schmidt and Wrisberg, 2008). Our results with larger gray matter volume and higher functional connectivity in basketball players involved in multiple cortical areas and various cortical networks suggest that complex motor and cognitive functions combining visual search, perceptual anticipation and action execution are required in the development of motor expertise (Abernethy, 1996; Vickers, 2004). It is not surprising that the gray matter volumes in motor related cortical areas increase and show enhanced connectivity with other cortical areas as frequent engagement of these cortical areas during long-term training induces the cortical plasticity in the underlying neuronal components and facilitates the communication of these components within the networks (Fries, 2005; Lewis et al., 2009; Duan et al., 2012). Interestingly, we found gray matter volume increases and functional connectivity enhanced in a wide range of cortical areas among three different functional networks in basketball players. Structural and functional changes in these areas largely contribute to the improvement of cognitive functions including temporal and spatial attention (Wright et al., 2013; Wu et al., 2013), memory processing (Wan et al., 2011; Wang et al., 2013), decision making and error correction (Koelewijn et al., 2008; Cocchi et al., 2013) in the population with professional experience. Our results are consistent with previous studies performed in other sports players (Di et al., 2012; Wang et al., 2013) and support the view that development of motor expertise relies on the improvement both in motor and cognitive functions (Aglioti et al., 2008). Future studies with further consideration about the interaction and mutual advantage between motor and cognitive components may help elucidate the mechanisms of cortical plasticity during the acquisition of high-level motor expertise.

We did not find increase in gray matter volume and functional connectivity related to primary motor cortex in basketball players compared to novices. This is consistent with previous studies in cohorts with other motor expertise, such as musicians (Fauvel et al., 2014), taxi drivers (Maguire et al., 2000) and athletes (Wei et al., 2011; Di et al., 2012; Wang et al., 2013). Interestingly, our previous studies with transcranial magnetic stimulation found increased motor cortical excitability during different motor tasks in athletes (Wang et al., 2014; Dai et al., 2016). It may be inferred that changes (both gray matter volume and functional connectivity) in other motor and cognitive related brain areas alter the cortico-cortical projections to the primary motor cortex and eventually lead to the increased output from the motor cortex in athletes. However, we cannot exclude the possibility that functional or even structural changes occur in the primary motor cortex itself after different courses of long-term motor training (Gaser and Schlaug, 2003; Draganski et al., 2004).

### Limitations

We investigated the morphological and functional differences between athletes and novices with a cross-sectional paradigm. It may be argued that larger gray matter volume and stronger functional connectivity observed in basketball players are not induced by long-term training but simply due to the natural property in this cohort which potentially leads to an “expert” brain with better structure and functions during development. Although our optimized VBM analysis partly ruled out the effect caused by inherent brain size difference in two groups, the question how cortical plasticity with long-term training is related to the structural and functional changes in the brain should be further addressed by longitudinal studies performed along the whole career of the athletes.

In addition, stronger connectivity in three functional networks was identified in the basketball players. It has long been controversial whether and how the resting-state functional connectivity represents the anatomical and biological connections in the brain (Raichle et al., 2001; Fox et al., 2005, 2006). Our study does not directly approach the question how the strong functional connectivity in the basketball players is related to the biological changes after the long-term training. However, our findings that functional connectivity was relevant to the seed region where structural (gray matter volume) difference was found between two groups and that we did not perform any pre-selection in determination of the seed region might suggest the potential biological changes with cortical plasticity after long-term training in athletes. The opinion is also consistent with the evidence obtained from neuroimaging studies performed in musicians that changes in functional map are often accompanied with structural changes during acquisition of motor expertise (Schlaug, 2001).

A reversed question is whether the result of functional connectivity analysis is affected by the difference in seed regions determined in the VBM approach. The findings that higher resting-state functional connectivity seen in basketball players compared to novices in the present study was based on the whole brain wide correlation analysis and that the cortical areas where higher functional connectivity were found (except for the seed regions) did not show greater gray matter volumes might partly deny the cause of increase in functional connectivity with simple changes in seed sizes. Similar increases both in gray matter volumes in seed regions and functional connectivity with the seed regions were also found in musicians (Fauvel et al., 2014). In addition, the reference time series in two subject groups are likely slightly different due to more gray matter volume in basketball players compared to novices when parametric approach is used in our time domain analysis (Friston et al., 1994). The comparison for functional connectivity between two groups may further be confounded by the partial volume effect around the seed regions (Müller-Gärtner et al., 1992). Although we performed additional masks in the seed regions by excluding voxels with low gray matter density (value below 0.3) to minimize partial volume effect, it may still be argued that the functional connectivity in novices is potentially underestimated with the fact that the selected seeds in the novices are contaminated by gray matter in other adjacent cortical areas or even white matter. The interaction between structural and functional changes during the long-term course of motor training is complex and the answer to this complex question again requires future work with longitudinal studies performed in the training course.

## Conclusion

Using structural and resting-state functional imaging techniques, the present study revealed larger volumes of gray matter in five seed regions and higher functional connectivity in default mode network, salience network and executive control network in basketball players compared to novices. We conclude that the morphology and functional connectivity in cortical neuronal networks in athletes and novices are different, and the differences may be related to higher level of motor expertise in athletes with better motor and cognitive functions.

## Author Contributions

X-YT and Y-LP conceived, designed and conducted experiments. YW analyzed data, interpreted results and wrote the manuscript. X-PL, L-LZ, WD and HZ helped conduct experiments and edited the manuscript. ZN, JW and JZ revised the manuscript. All authors approved the submitted version.

## Funding

The present study was funded by the National Natural Science Foundation of China (No. 31470051, No. 31371056), Shanghai Pudong New Area Health Bureau (No. PWZxkq 2011-02) and Shanghai Key Lab of Human Performance (Shanghai University of Sport, No. 11DZ2261100).

## Conflict of Interest Statement

The authors declare that the research was conducted in the absence of any commercial or financial relationships that could be construed as a potential conflict of interest.

## Footnotes

^1^http://www.fil.ion.ucl.ac.uk/spm/

^2^http://www.nitrc.org/projects/bnv/
